# Genome-wide Association Study for Carcass Primal Cut Yields Using Single-step Bayesian Approach in Hanwoo Cattle

**DOI:** 10.3389/fgene.2021.752424

**Published:** 2021-11-26

**Authors:** Masoumeh Naserkheil, Hossein Mehrban, Deukmin Lee, Mi Na Park

**Affiliations:** ^1^ Animal Breeding and Genetics Division, National Institute of Animal Science, Cheonan-si, South Korea; ^2^ Department of Animal Science, Shahrekord University, Shahrekord, Iran; ^3^ Department of Animal Life and Environment Sciences, Hankyong National University, Anseong-si, South Korea

**Keywords:** candidate genes, QTL, carcass primal cut yield, single-step GWAS, hanwoo

## Abstract

The importance of meat and carcass quality is growing in beef cattle production to meet both producer and consumer demands. Primal cut yields, which reflect the body compositions of carcass, could determine the carcass grade and, consequently, command premium prices. Despite its importance, there have been few genome-wide association studies on these traits. This study aimed to identify genomic regions and putative candidate genes related to 10 primal cut traits, including tenderloin, sirloin, striploin, chuck, brisket, top round, bottom round, shank, flank, and rib in Hanwoo cattle using a single-step Bayesian regression (ssBR) approach. After genomic data quality control, 43,987 SNPs from 3,745 genotyped animals were available, of which 3,467 had phenotypic records for the analyzed traits. A total of 16 significant genomic regions (1-Mb window) were identified, of which five large-effect quantitative trait loci (QTLs) located on chromosomes 6 at 38–39 Mb, 11 at 21–22 Mb, 14 at 6–7 Mb and 26–27 Mb, and 19 at 26–27 Mb were associated with more than one trait, while the remaining 11 QTLs were trait-specific. These significant regions were harbored by 154 genes, among which *TOX*, *FAM184B*, *SPP1*, *IBSP*, *PKD2*, *SDCBP*, *PIGY*, *LCORL*, *NCAPG*, and *ABCG2* were noteworthy. Enrichment analysis revealed biological processes and functional terms involved in growth and lipid metabolism, such as growth (GO:0040007), muscle structure development (GO:0061061), skeletal system development (GO:0001501), animal organ development (GO:0048513), lipid metabolic process (GO:0006629), response to lipid (GO:0033993), metabolic pathways (bta01100), focal adhesion (bta04510), ECM–receptor interaction (bta04512), fat digestion and absorption (bta04975), and Rap1 signaling pathway (bta04015) being the most significant for the carcass primal cut traits. Thus, identification of quantitative trait loci regions and plausible candidate genes will aid in a better understanding of the genetic and biological mechanisms regulating carcass primal cut yields.

## Introduction

Hanwoo is an indigenous and popular meat-type cattle in Korea, and is particularly renowned for its rapid growth rate and quality attributes such as juiciness, tenderness, characteristic flavor, and extensive marbling of its beef (Jo et al., 2012). In recent years, both carcass and meat quality traits in Hanwoo have been extensively studied because of their economic relevance for optimizing the profitability of the beef industry. The current selection index in Hanwoo focuses on the improvement of carcass traits, such as backfat thickness (BFT), carcass weight (CW), eye muscle area (EMA), and marbling score (MS) as major selection criteria for breeding programs (Kim et al., 2017). However, other important traits such as carcass primal cuts have received inadequate attention in the Hanwoo breeding program, which affects both the quantity and quality of meat, and consequently, command premium prices. To meet consumer demand, the importance of primal cut yields is growing in the beef industry of developed countries because of its market value. Hence, cattle breeders need to address these traits, which determine selection decisions to increase carcass cut-out value and consumer acceptance of meat. Meanwhile, the existence of genetic variation and moderate to high heritability in the yield of primal cuts has been reported (Choi et al., 2015). In this sense, the improvement of primal cut yields requires knowledge of the underlying genetic background influencing these invaluable traits.

Over the last decade, with the development of high-throughput single nucleotide polymorphism (SNP) genotyping technologies, genome-wide association studies (GWAS) have become an affordable and powerful tool for detecting and localizing candidate genes and causal mutations associated with quantitative traits in different species (Matukumalli et al., 2009). Several statistical methods to conduct GWAS have been developed and applied, among which a simple regression model has been widely used, where one marker is tested at a time for significance (Meyer and Tier, 2012). However, this method was challenged by false positives and overestimation of quantitative trait loci (QTL) effects. Therefore, the marker effect models in the Bayesian approaches have been proposed for GWAS analysis (Habier et al., 2011; Wang et al., 2012; Moser et al., 2015; Wang et al., 2016) as they offer methods to overcome these challenges (Strömberg, 2009; Peters et al., 2012). One such method could have a higher power to detect SNPs with moderate effects on a trait of interest. In addition, Bayesian methods are flexible in accounting for the uncertainties of variables and parameters and allow for inferences to be made by finding their marginal posterior distributions (Yi and Shriner, 2008; Blasco and Blasco, 2017). Recently, Fernando et al. (2014; 2016) developed a class of single-step Bayesian regression methods (ssBR), which not only combines all available information as single-step genomic best linear unbiased prediction [ssGBLUP; (Misztal et al., 2009)] does, but also accommodates Bayesian models. This method can also be extended to GWAS and controls the proportion of false positives by computing the posterior probability of association of a trait with each SNP or each window of consecutive SNPs. Numerous association studies have been carried out on growth and carcass traits in beef cattle using different GWAS approaches (Peters et al., 2012; Lee et al., 2013; Magalhaes et al., 2016; Weng et al., 2016; Roberts, 2018; Bedhane et al., 2019; Naserkheil et al., 2020). However, GWAS have not yet been conducted to identify significant genomic regions for carcass primal cut traits, which are highly relevant to Hanwoo cattle breeding. Hence, the objective of this study was to perform GWAS to detect genomic regions and candidate genes associated with primal cut yields in Hanwoo cattle using the ssBR approach.

## Materials and Methods

### Animal and Phenotype Data

A total of 3,467 Hanwoo steers born between 2008 and 2017 were included in this study. All steers were slaughtered at approximately 24 months of age and were progeny of 442 sires and 3,357 dams. The pedigree data consisted of 15,117 animals after tracing the pedigree file back 10 generations and pruning with SECATEURS software (Meyer, 2003). During the pruning process, 3,692 individuals were removed from the pedigree. All phenotypic data were collected by the Hanwoo Improvement Center (HIC) of the National Agricultural Cooperative Federation, South Korea. The ten primal cut yields considered in the present study (kg, composed of both unique and composite meat cuts from the forequarters and hindquarters) included tenderloin, sirloin, striploin, chuck, brisket, top round, bottom round, shank, flank, and rib; the locations of each cut on the carcass are illustrated in Supplementary Figure S1. The descriptive statistics and heritability estimates of the primal cut traits are shown in Table 1.

**TABLE 1:** Descriptive statistics and heritability estimates for the primal cut traits in Hanwoo cattle.

Trait (unit)	No. of records	Mean (SE)	Min	Max	SD	CV (%)	h^2^ (SD)
Tenderloin (Kg)	3,466	6.04 (0.01)	3	9	0.76	12.65	0.47 (0.03)
Sirloin (Kg)	3,465	34.23 (0.07)	16.8	50.7	4.11	12.02	0.49 (0.04)
Striploin (Kg)	3,465	7.85 (0.02)	4.3	12.4	1.17	14.96	0.46 (0.04)
Chuck (Kg)	3,463	14.61 (0.06)	6.7	34.8	3.76	25.72	0.32 (0.03)
Brisket (Kg)	3,466	23.76 (0.05)	12.6	38.6	3.01	12.67	0.59 (0.04)
Top round (Kg)	3,467	20.22 (0.04)	10.5	30.2	2.43	12	0.58 (0.04)
Bottom round (Kg)	3,467	32.99 (0.07)	16.6	49.6	3.92	11.89	0.58 (0.03)
Shank (Kg)	3,466	14.66 (0.03)	9	21.7	1.77	12.09	0.61 (0.04)
Flank (Kg)	3,465	28.29 (0.08)	12.5	50.3	4.83	17.08	0.35 (0.03)
Rib (Kg)	3,467	57.55 (0.13)	21.7	89.3	7.53	13.09	0.40 (0.04)

SE, standard error; SD, standard deviation; CV, coefficient of variation; h^2^, heritability.

### Genotype Data

In total, 12,764 animals were genotyped initially using three different SNP platforms, Illumina BovineSNP50K version 2 (*n* = 3,720), version 3 (*n* = 4,121), and customized Hanwoo version 1 (*n* = 4,923). Individuals (and SNPs) with a call rate of less than 90% and those without a valid phenotype were also excluded. The genotyped animals with Illumina BovineSNP50K version 2 were used as a reference populations to impute target animals (The genotyped animals using Illumina BovineSNP50K version 3 and customized Hanwoo version 1) using FImpute V3 software (Sargolzaei et al., 2014), and 52,791 SNPs on the 29 chromosomes were finally obtained. The analyses included genotypes for 2,957 steers with phenotypes and 788 their paternal ancestors. Quality control procedures were conducted using the BLUPF90 software (Misztal et al., 2015). SNPs with minor allele frequency less than 0.01 (8,783 SNPs), and a maximum difference between the observed and expected frequency of 0.15 as a departure of heterozygous from the Hardy-Weinberg equilibrium (21 SNPs) were discarded. After quality control, 3,745 animals with the genotypes on 43,987 SNPs were remained for subsequent analyses.

### Association Analyses

The single-step Bayesian regression (ssBR) method proposed by Fernando et al. (2014; 2016) was utilized to perform GWAS analyses, which combined all available phenotypes, genotypes, and pedigree information in a single-step. The estimation of genetic and residual variances as well as GWAS analyses were performed using univariate single-step Bayes B, with π being 0.99. The model for the single-step Bayesian GWAS (Fernando et al., 2014; Cheng et al., 2015; Fernando et al., 2016; Fernando et al., 2017) was as follows for genotyped animals:

$$y_{i}=\sum_{\mathrm{j=1}}^{p_{\beta }}X_{\mathrm{ij}}\beta_{j}+\sum_{\mathrm{k=1}}^{P_{u}}Z_{\mathrm{ik}}u_{k}+\sum_{\mathrm{l=1}}^{P}M_{\mathrm{il}}\alpha_{l}+e_{i}$$ (1)

and non-genotyped animals:

$$y_{i}=\sum_{\mathrm{j=1}}^{P_{\beta }}X_{\mathrm{ij}}\beta_{j}+\sum_{\mathrm{k=1}}^{P_{u}}Z_{\mathrm{ik}}u_{k}+\sum_{\mathrm{l=1}}^{p}{\hat{M}}_{\mathrm{il}}\alpha_{l}+\sum_{\mathrm{m=1}}^{p_{\varepsilon }}Z_{\mathrm{n[i,m]}}\varepsilon_{m}+e_{i}$$ (2)

where y_i_ is a phenotype for individual i; β_j_ is the *j*th effects including slaughter date (180 levels) and slaughter age (days from birth to slaughter) was considered as covariates; X_ij_ is the incidence covariate corresponding to the β_j_ for individual i; Z_ik_ is the incidence covariate corresponding to the *k*th random animal effect for individual i; **u** = [u1, u2, …, ${\text{u}}_{{\text{p}}_{\text{u}}}$ ] is the vector of random animal effect assumed normally distributed N (**0**, **A** σ_u_^2^), **A** is the numerator relationship matrix and σ_u_^2^ is additive genetic variance; M_il_ is the genotype covariate (coded as 0,1,2) at locus l for individual i; ${\hat{\text{M}}}_{\text{il}}$ is the imputed genotype covariate at locus l for non-genotyped individual i; α_l_ is the allele substitution effect or marker effect for locus l assumed t-Student distributed t (0, σ_α_^2^) with probability 1- π and zero with π = 0.99, σ_α_^2^ is marker variance; Z_n[i,m]_ is the incidence covariate corresponding to the *m*th imputation residual for individual i and ϵ_m_ is the imputation residual; *p* is the number of genotyped loci; ${\text{p}}_{\text{β}}$ is the number of effect levels for **β**; ${\text{p}}_{\text{u}}$ is the number of random animal effect levels; $p_{\varepsilon }$ is the number of non-genotyped animals; and e_i_ is the *i*th random residual effect for individual i assumed normally distributed N (0, σ_e_^2^), and σ_e_^2^ is residual variance.

The effects of **β** are assigned to the flat priors. In addition, the additive genetic (σ_u_^2^), residual (σ_e_^2^), and marker (σ_α_^2^) variances were assumed to have a scaled inverted chi-square prior with scale parameters $S_{\alpha }^{2}$ and ν_α_ degrees of freedom. The prior means for additive genetic and residual variances were estimated using an animal model. In addition, the prior means was equal to ${\text{σ}}_{\text{u}}^{2}/\left[\left(1-\text{π}\right)\overset{\text{p}}{\underset{\text{l}=1}{\sum }}2{\text{p}}_{\text{l}}\left(1-{\text{p}}_{\text{l}}\right)\right]$ for marker variance, as proposed by Habier et al. (2011), where p_i_ is the allelic frequency at the *l*th locus. The degrees of freedom were four for residual and marker variances, and five for additive genetic variance.

The analysis was performed using the JWAS Julia package for whole-genome analyses (Cheng et al., 2018) to obtain the posterior distributions of SNP effects using Markov chain Monte Carlo (MCMC). This method with 110,000 iterations was implemented to provide the posterior mean effects of the SNPs within each 1-Mb window and variance components after discarding the first 10,000 samples for burn-in and a thinning interval of 10. In total, 2,522 windows (1-Mb) across the 29 autosomes were included in the analyses. The window posterior probabilities of association (WPPA) for each window were also calculated.

The markers effect in each MCMC was estimated by using single-step Bayes B in addition to the polygenic additive genetic variance (σ^2^_a_) and residual variance (σ^2^_e_). The direct genomic value (DGV) that is attributed to markers is estimated as:

$$DGV_{i}=\overset{p}{\underset{l=1}{\sum }}M_{il}\alpha_{l}\;\;$$

The genomic variance (σ^2^_m_) was estimated using Gibbs sampling technique described by Sorensen et al. (2001). Then total genetic variance was estimated by summation of σ^2^_m_ and σ^2^_u_. In addition, the phenotypic variance was estimated by summation total genetic and environment variances. The heritability was obtained using total genetic variance divided by phenotypic variance.

The percentage of genomic variance (GV%) explained by each 1-Mb window in any particular iteration was calculated by dividing the genomic variance of the window by the genomic variance of the whole genome in the same iteration. Similarity, the proportion of additive genetic variance (AGV%) determined using each window markers were also obtained.

### Identification of Candidate Genes and Functional Enrichment Analysis

Genome windows with WPPA ≥0.8 (Wang et al., 2020) were considered as possible QTL regions associated with the studied traits. Candidate genes were searched for 1-Mb window using the Ensembl database and the Map Viewer tool of the bovine genome based on the starting and ending coordinates of significant windows. Further information on the function of these genes was obtained from the National Center for Biotechnology Information (NCBI) (http://www.ncbi.nlm.nih.gov/gene/), and GeneCards (www.genecards.org). A Manhattan plot was created using the ggplot2 package (Wickham, 2009) in R software. To understand and identify the biological processes and pathways, gene ontology (GO) and Kyoto Encyclopedia of Genes and Genomes (KEGG) enrichment were carried out using the Database for Annotation, Visualization, and Integrated Discovery (DAVID) and web tool g:Profiler (Raudvere et al., 2019). Only GO terms with a significant *p* value of 0.05 and genes involved in biological processes, molecular functions, and cellular components were highlighted.

## Results

The number of records, means, minimum, maximum, standard deviations, phenotypic coefficient of variation, and heritability estimates for the 10 primal cut traits are provided in Table 1. The mean values of these traits ranged from 6.04 to 57.55 with standard deviation between 0.76 and 7.53. The coefficients of variation ranged from 11.89 to 25.72%, indicating considerable phenotypic variation of the investigated traits in the Hanwoo cattle population. Moderate to high heritability estimates were obtained for primal cut yields, which ranged from 0.32 ± 0.03 to 0.61 ± 0.04. Estimated variance components are also summarized in Supplementary Table S1.

**FIGURE 1:**
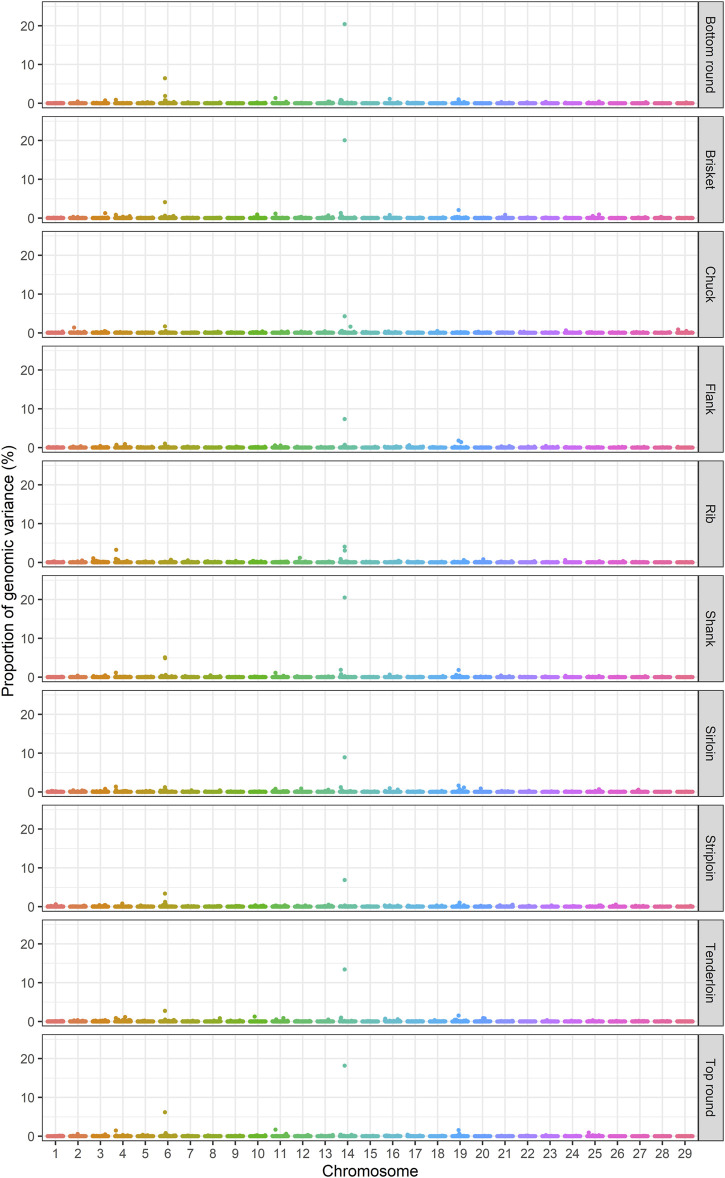
Manhattan plots of the percentage of genomic variance explained by 1-Mb windows for primal cut traits in Hanwoo cattle.

**FIGURE 2:**
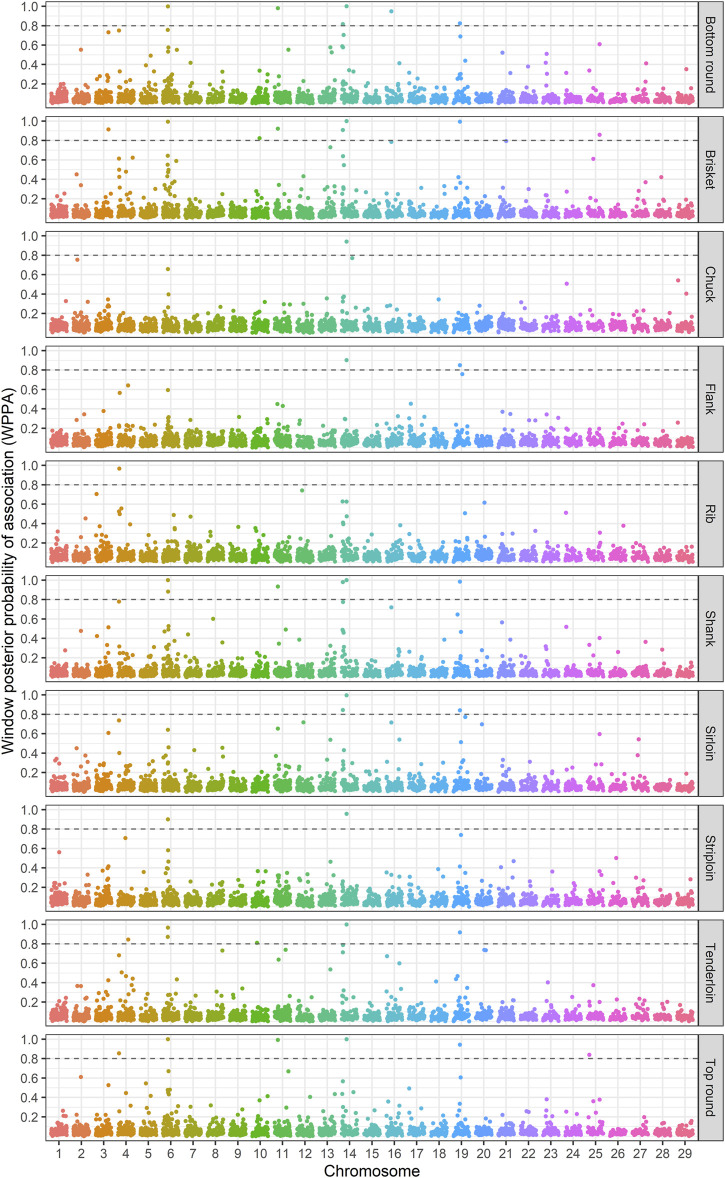
Plots of window posterior probabilities of association (WPPA) obtained by the single-step Bayesian regression method for primal cut traits in Hanwoo cattle. The dash line is threshold 0.8 for significantly of windows.

The identification of genomic regions related to the traits of interest was performed using a ssBR approach. Only windows with WPPA ≥0.8, were considered significant. Manhattan plots displayed the proportion of genomic variance (Figure 1), additive genetic variance (Supplementary Figure S2) explained and the posterior probability of association by each 1-Mb window for the studied traits (Figure 2). Positional candidate genes for primal cut traits were also detected within the significant windows. A summary of significant windows associated with the traits under study, such as the number of SNPs in each window, explained GV% or AGV%, and WPPA, as well as candidate genes, is shown in Table 2. A total of 16 relevant genomic regions (1-Mb window) were found to be associated with the 10 traits recorded in this study. These regions were distributed over nine different chromosomes: 3, 4, 6, 10, 11, 14, 16, 19, and 25. Of these significant windows, five genomic windows were pleiotropic QTL, meaning that the QTL had an effect on multiple traits, which were located on chromosomes 6 at 38–39, 11 at 21–22, 14 at 6–7 Mb and 26–27, and 19 at 26–27 Mb. The genomic window located on chromosome 14 at 26–27 Mb explained a large proportion of the genomic variance across the nine analyzed traits, including tenderloin, sirloin, striploin, chuck, brisket, top round, bottom round, shank, and flank. The largest QTL window was observed for shank, which explained 20.51% of the genomic variance, was located in the region of 26–27 Mb on chromosome 14. The QTL window with the smallest proportion of genomic variance (0.87%) was identified for bottom round, located on chromosome 14 at 6–7 Mb. Candidate genes responsible for the genomic variance explained by the 1-Mb window were identified using the *Bos taurus* genome map. A total of 154 genes were identified within the significant regions to be associated with the traits of interest. Of these, 92 genes were codified proteins, 3 were miRNA (microRNA), 6 were tRNA (RNA transporter), and 53 were pseudogenes (Table 2). Functional enrichment analysis revealed 18 biological processes, six molecular functions, seven cellular components, and seven KEGG pathways. The following biological process terms were highlighted: growth (GO:0040007), positive regulation of growth (GO:0045927), tissue development (GO:0009888), muscle structure development (GO:0061061), skeletal system development (GO:0001501), animal organ development (GO:0048513), cellular response to chemical stimulus (GO:0070887), lipid metabolic process (GO:0006629), response to lipid (GO:0033993), glycolipid biosynthetic process (GO:0009247), and cell cycle (GO:0007049) being the most significant for the traits under study. In the case of the molecular function, calcium channel activity (GO:0005262), protein binding (GO:0005515), ligase activity (GO:0016874), lipid binding (GO:0008289), catalytic activity (GO:0003824), and nucleotide binding (GO:0000166) were significant terms. The significant enrichments for cellular component, including endoplasmic reticulum membrane (GO:0005789), intracellular (GO:0005622), endoplasmic reticulum (GO:0005783), actin cytoskeleton (GO:0015629), extracellular region (GO:0005576), organelle (GO:0043226), and cytoplasmic region (GO:0099568) were obtained. Moreover, KEGG pathway analysis revealed that the identified candidate genes involved in primal cut yields were enriched in metabolic pathways (bta01100), focal adhesion (bta04510), ECM-receptor interaction (bta04512), glycolysis/gluconeogenesis (bta00010), fat digestion and absorption (bta04975), Rap1 signaling pathway (bta04015), and calcium signaling pathway (bta04020). Functional gene set annotation and enrichment pathways are presented in Table 3.

**TABLE 2:** Gene identification and proportion of variance explained by 1-Mb windows associated with the primal cut traits in Hanwoo cattle.

Trait	Chr	QTL region (Mb)	Number of SNPs	GV%	AGV%	WPPA	Candidate genes
Tenderloin	14	26–27	24	13.39	9.93	0.99	FAM110B, LOC101902490, UBXN2B, CYP7A1, TRNAG-CCC, SDCBP, NSMAF, LOC101902713, LOC107133116, TOX, TRNAC-GCA
Tenderloin	6	38–39	19	2.7	2.00	0.96	PKD2, SPP1, MEPE, IBSP, LOC104972726, TRNAA-CGC, LAP3, MED28, FAM184B, NCAPG, DCAF16, LCORL
Tenderloin	19	26–27	21	1.52	1.12	0.91	PITPNM3, FAM64A, AIPL1, WSCD1, LOC104975014, NLRP1, LOC788205, MIS12, DERL2, DHX33, C1QBP, RPAIN, NUP88, MIR199C, RABEP1, LOC101904050, SCIMP, LOC107131511
Tenderloin	6	37–38	25	2.74	2.04	0.87	LOC104972722, TIGD2, FAM13A, LOC104972723, LOC104972724, LOC100847719, HERC3, NAP1L5, PYURF, PIGY, HERC5, HERC6, PPM1K, ABCG2, LOC781421
Tenderloin	4	77–78	18	1.13	0.84	0.84	RAMP3, WAP, TBRG4, NACAD, CCM2, LOC100140586, LOC101904529, MYO1G, PURB, MIR4657, H2AFV, PPIA, ZMIZ2, OGDH, TMED4, DDX56, LOC104972145, NPC1L1, NUDCD3, LOC104972146, CAMK2B, YKT6, GCK, MYL7, POLD2, AEBP1, POLM
Tenderloin	10	32–33	20	1.24	0.92	0.81	C10H15orf41, LOC107131394, LOC101904022, LOC104973117, MEIS2, LOC104973118
Sirloin	14	26–27	24	8.93	5.88	0.99	FAM110B, LOC101902490, UBXN2B, CYP7A1, TRNAG-CCC, SDCBP, NSMAF, LOC101902713, LOC107133116, TOX, TRNAC-GCA
Sirloin	14	6–7	33	1.23	0.81	0.84	
Sirloin	19	26–27	21	1.62	1.07	0.84	PITPNM3, FAM64A, AIPL1, WSCD1, LOC104975014, NLRP1, LOC788205, MIS12, DERL2, DHX33, C1QBP, RPAIN, NUP88, MIR199C, RABEP1, LOC101904050, SCIMP, LOC107131511
Striploin	14	26–27	24	6.86	4.62	0.95	FAM110B, LOC101902490, UBXN2B, CYP7A1, TRNAG-CCC, SDCBP, NSMAF, LOC101902713, LOC107133116, TOX, TRNAC-GCA
Striploin	6	38–39	19	3.40	2.29	0.90	PKD2, SPP1, MEPE, IBSP, LOC104972726, TRNAA-CGC, LAP3, MED28, FAM184B, NCAPG, DCAF16, LCORL
Chuck	14	26–27	24	4.26	3.06	0.94	FAM110B, LOC101902490, UBXN2B, CYP7A1, TRNAG-CCC, SDCBP, NSMAF, LOC101902713, LOC107133116, TOX, TRNAC-GCA
Brisket	14	26–27	24	20.03	13.67	1.00	FAM110B, LOC101902490, UBXN2B, CYP7A1, TRNAG-CCC, SDCBP, NSMAF, LOC101902713, LOC107133116, TOX, TRNAC-GCA
Brisket	6	38–39	19	4.11	2.80	0.99	PKD2, SPP1, MEPE, IBSP, LOC104972726, TRNAA-CGC, LAP3, MED28, FAM184B, NCAPG, DCAF16, LCORL
Brisket	19	26–27	21	2.07	1.41	0.99	PITPNM3, FAM64A, AIPL1, WSCD1, LOC104975014, NLRP1, LOC788205, MIS12, DERL2, DHX33, C1QBP, RPAIN, NUP88, MIR199C, RABEP1, LOC101904050, SCIMP, LOC107131511
Brisket	11	21–22	27	1.15	0.79	0.92	GALM, SRSF7, GEMIN6, LOC107132913, DHX57, MORN2, ARHGEF33, SOS1, MIR2284Z-2, LOC104973309, CDKL4, LOC782845, MAP4K3, LOC107132914, TMEM178A
Brisket	3	98–99	24	1.27	0.87	0.91	AGBL4, BEND5, LOC104971807, SPATA6, TRNAT-AGU, LOC107132336, SLC5A9, LOC107131387, SKINT1, TRNAR-ACG, LOC101906301, TRABD2B
Brisket	14	6–7	33	1.31	0.89	0.90	
Brisket	25	32–33	19	0.92	0.63	0.85	LOC104975903, LOC104975897, RN18S1, LOC104975899, LOC107131836, LOC107131838, LOC107131837, LOC107131839
Brisket	10	49–50	24	0.92	0.63	0.82	RORA, LOC107132858, LOC107132859, LOC104973153, ICE2, LOC107132852, LOC101902861, ANXA2
Top round	14	26–27	24	18.18	12.40	1.00	FAM110B, LOC101902490, UBXN2B, CYP7A1, TRNAG-CCC, SDCBP, NSMAF, LOC101902713, LOC107133116, TOX, TRNAC-GCA
Top round	6	38–39	19	6.19	4.22	0.99	PKD2, SPP1, MEPE, IBSP, LOC104972726, TRNAA-CGC, LAP3, MED28, FAM184B, NCAPG, DCAF16, LCORL
Top round	11	21–22	27	1.69	1.15	0.99	GALM, SRSF7, GEMIN6, LOC107132913, DHX57, MORN2, ARHGEF33, SOS1, MIR2284Z-2, LOC104973309, CDKL4, LOC782845, MAP4K3, LOC107132914, TMEM178A
Top round	19	26–27	21	1.57	1.07	0.94	PITPNM3, FAM64A, AIPL1, WSCD1, LOC104975014, NLRP1, LOC788205, MIS12, DERL2, DHX33, C1QBP, RPAIN, NUP88, MIR199C, RABEP1, LOC101904050, SCIMP, LOC107131511
Top round	4	6–7	14	1.46	1.00	0.85	LOC101904266, LOC104970217, LOC781773, LOC107132367
Top round	25	4–5	25	0.93	0.64	0.83	SEC14L5, NAGPA, C25H16orf89, ALG1, LOC101905725, EEF2KMT, LOC521021, LOC107131809, LOC104975833
Bottom round	14	26–27	24	20.42	15.21	1.00	FAM110B, LOC101902490, UBXN2B, CYP7A1, TRNAG-CCC, SDCBP, NSMAF, LOC101902713, LOC107133116, TOX, TRNAC-GCA
Bottom round	6	38–39	19	6.46	4.83	0.99	PKD2, SPP1, MEPE, IBSP, LOC104972726, TRNAA-CGC, LAP3, MED28, FAM184B, NCAPG, DCAF16, LCORL
Bottom round	11	21–22	27	1.36	1.02	0.98	GALM, SRSF7, GEMIN6, LOC107132913, DHX57, MORN2, ARHGEF33, SOS1, MIR2284Z-2, LOC104973309, CDKL4, LOC782845, MAP4K3, LOC107132914, TMEM178A
Bottom round	16	25–26	8	1.13	0.84	0.94	HLX, TRNAQ-CUG, DUSP10
Bottom round	19	26–27	21	1.00	0.75	0.82	PITPNM3, FAM64A, AIPL1, WSCD1, LOC104975014, NLRP1, LOC788205, MIS12, DERL2, DHX33, C1QBP, RPAIN, NUP88, MIR199C, RABEP1, LOC101904050, SCIMP, LOC107131511
Bottom round	14	6–7	33	0.87	0.65	0.81	
Shank	14	26–27	24	20.51	14.02	1.00	FAM110B, LOC101902490, UBXN2B, CYP7A1, TRNAG-CCC, SDCBP, NSMAF, LOC101902713, LOC107133116, TOX, TRNAC-GCA
Shank	6	38–39	19	5.09	3.48	0.99	PKD2, SPP1, MEPE, IBSP, LOC104972726, TRNAA-CGC, LAP3, MED28, FAM184B, NCAPG, DCAF16, LCORL
Shank	19	26–27	21	1.86	1.27	0.98	PITPNM3, FAM64A, AIPL1, WSCD1, LOC104975014, NLRP1, LOC788205, MIS12, DERL2, DHX33, C1QBP, RPAIN, NUP88, MIR199C, RABEP1, LOC101904050, SCIMP, LOC107131511
Shank	14	6–7	33	1.90	1.30	0.98	
Shank	11	21–22	27	1.13	0.77	0.93	GALM, SRSF7, GEMIN6, LOC107132913, DHX57, MORN2, ARHGEF33, SOS1, MIR2284Z-2, LOC104973309, CDKL4, LOC782845, MAP4K3, LOC107132914, TMEM178A
Shank	6	39–40	24	4.89	3.35	0.88	LOC782905
Flank	14	26–27	24	7.37	5.34	0.90	FAM110B, LOC101902490, UBXN2B, CYP7A1, TRNAG-CCC, SDCBP, NSMAF, LOC101902713, LOC107133116, TOX, TRNAC-GCA
Flank	19	26–27	21	1.80	1.30	0.84	PITPNM3, FAM64A, AIPL1, WSCD1, LOC104975014, NLRP1, LOC788205, MIS12, DERL2, DHX33, C1QBP, RPAIN, NUP88, MIR199C, RABEP1, LOC101904050, SCIMP, LOC107131511
Rib	4	8–9	18	3.23	2.26	0.96	CDK14, LOC104971924, FZD1, LOC782091, LOC100140224

GV%, proportion of the genomic variance explained by window; AGV%, proportion of the additive genetic variance explained by window; WPPA, window posterior probability of association. *Table was decreasingly sorted based on the WPPA, within each trait.

**TABLE 3:** Gene Ontology (GO) terms and KEGG pathways significantly enriched using candidate genes associated with the primal cut traits.

Term ID	Term name	Count	Genes	*p*-value
Biological process				
GO:0040007	Growth	5	CCM2, HLX, SOS1, DERL2, SDCBP	5.40E-06
GO:0045927	positive regulation of growth	3	HLX, DERL2, SDCBP	1.50E-04
GO:0007517	muscle organ development	2	HLX, FZD1	1.80E-02
GO:0009888	tissue development	10	PKD2, SPP1, MEPE, IBSP, CCM2, HLX, SOS1, ANXA2, FZD1, SDCBP	6.20E-10
GO:0061061	muscle structure development	4	HLX, FZD1, RORA, MED28	2.80E-05
GO:0060284	regulation of cell development	4	CAMK2B, C1QBP, DUSP10, SDCBP	8.30E-05
GO:0001501	skeletal system development	2	ANXA2, MEPE	2.70E-02
GO:0031214	biomineral tissue development	4	ANXA2, MEPE, SPP1, IBSP	3.70E-05
GO:0048513	animal organ development	16	CCM2, HLX, SOS1, SDCBP, POLM, HERC6, MEIS2, RORA, ANXA2, FZD1, IBSP, MEPE, OGDH, PKD2, SPP1, TMEM178A	1.20E-11
GO:0006629	lipid metabolic process	5	NPC1L1, PYURF, PIGY, RORA, CYP7A1	1.60E-05
GO:0033993	response to lipid	4	RORA, DUSP10, RAMP3, SPP1	3.10E-05
GO:0042157	lipoprotein metabolic process	3	NPC1L1, PYURF, PIGY	5.40E-05
GO:0006096	glycolytic process	2	GCK, OGDH	3.30E-03
GO:0009247	glycolipid biosynthetic process	2	PYURF, PIGY	7.70E-03
GO:0045598	regulation of fat cell differentiation	2	RORA, DUSP10	6.70E-03
GO:0051179	localization	6	MIS12, PITPNM3, C1QBP, DERL2, NUP88, RABEP1	2.40E-03
GO:0070887	cellular response to chemical stimulus	9	RORA, C1QBP, CYP7A1, DERL2, FZD1, IBSP, PKD2, RAMP3, SDCBP	4.30E-08
GO:0007049	cell cycle	4	MIS12, NCAPG, PKD2, SDCBP	4.20E-04
Molecular Function				
GO:0005262	calcium channel activity	2	ANXA2, PKD2	3.12E-02
GO:0005515	protein binding	4	SPP1, MEPE, PKD2, MED28	4.76E-02
GO:0016874	Ligase activity	3	HERC5, HERC3, HERC6	2.76E-03
GO:0008289	lipid binding	2	ANXA2, RORA	3.52E-02
GO:0003824	catalytic activity	5	HERC5, HERC3, PPM1K, ABCG2, HERC6	7.40E-03
GO:0000166	nucleotide binding	4	CDKL4, DHX57, SRSF7, MAP4K3	5.10E-03
Cellular Component				
GO:0005789	endoplasmic reticulum membrane	8	CAMK2B, PYURF, TMEM178A, DERL2, PKD2, CYP7A1, PIGY, TMED4	1.30E-03
GO:0005622	Intracellular	8	HERC5, PYURF, HERC3, PPM1K, PIGY, NAP1L5, ABCG2, HERC6	3.00E-02
GO:0005783	endoplasmic reticulum	8	CAMK2B, PYURF, TMEM178A, DERL2, ALG1, PKD2, CYP7A1, PIGY	8.30E-03
GO:0015629	actin cytoskeleton	3	NCAPG, PKD2, MED28	1.10E-02
GO:0005576	extracellular region	5	IBSP, SPP1, MEPE, LAP3, PKD2	4.52E-02
GO:0043226	Organelle	8	HERC5, PYURF, HERC3, PPM1K, PIGY, NAP1L5, ABCG2, HERC6	4.83E-02
GO:0099568	cytoplasmic region	2	PKD2, MED28	3.50E-02
KEGG pathways				
bta01100	Metabolic pathways	8	POLD2, OGDH, GALM, LAP3, ALG1, CYP7A1, PIGY, GCK	5.03E-07
bta04512	ECM -receptor interaction	2	SPP1, IBSP	3.80E-03
bta04510	Focal adhesion	4	SPP1, IBSP, MYL7, SOS1	2.10E-05
bta04975	Fat digestion and absorption	1	NPC1L1	8.20E-05
bta04020	Calcium signaling pathway	1	CAMK2B	7.10E-03
bta00010	Glycolysis/Gluconeogenesis	2	GALM, GCK	8.30E-03
bta04015	Rap1 signaling pathway	1	LCP2	3.30E-03

## Discussion

The aim of this study was to identify genomic regions associated with primal cut yields using a ssBR approach in Hanwoo cattle. The marker effect model in a Bayesian framework would seem to be useful for GWAS because they account for uncertainty in parameters required to construct posterior distributions for QTL inference, thereby improving accuracy of genomic predictions and the power of QTL detection (Fernando and Garrick, 2013; Mehrban et al., 2017). In recent years, interest in exploring genomic regions that control economically important traits in beef cattle has increased due to advances in high-throughput genotyping techniques and the constant availability of molecular data, statistical methods, and ease of application of GWAS. Primal cut traits have recently been proposed as potential indicator of carcass weight and overall carcass merit (Berry et al., 2019) given that the genetic correlations between these traits and carcass weight are generally moderate to strong (Choi et al., 2015; Judge et al., 2019). Nevertheless, selection for the weight of primal cuts requires knowledge of the genetic basis for these traits, which may be useful in future genomic evaluations targeting the improvement of weight in the more valuable primal cuts, and consequently increasing the profitability of the meat production system. Our results showed that primal cut yields were moderate to highly heritable, being in accordance with those reported in Hanwoo cattle (Choi et al., 2015), Chianina cattle (Sarti et al., 2013), Simmental cattle (Zhu et al., 2019), and Irish cattle (Berry et al., 2019; Judge et al., 2019). Among all identified window regions, the QTL on chromosome 14 at position 26–27 Mb had a larger impact than any of the other QTLs and was associated with a greater number of traits. This region, which is related to tenderloin, sirloin, striploin, chuck, brisket, top round, bottom round, shank, and flank, explained between 4.26 and 20.51% of the genomic variance across all these traits. A total of 11 genes were detected on chromosome 14 at 26–27 Mb regions. Among these, *FAM110B*, *UBXN2B*, *NSMAF*, *TOX*, *SDCBP*, and *CYP7A1* were notable. This region also seems to be most significantly associated with carcass and growth traits in beef cattle (Magalhaes et al., 2016; Roberts, 2018; Zhang et al., 2019; Grigoletto et al., 2020). Positional candidate genes of *FAM110B*, *UBXN2B*, *NSMAF*, *CYP7A1*, *SDCBP*, and *TOX* have been previously reported to be associated with carcass weight in Hanwoo cattle (Lee et al., 2013; Bhuiyan et al., 2018; Naserkheil et al., 2020). For instance, Lee et al. (2013) identified the six most significant SNPs associated with carcass weight in Hanwoo that were located in or nearby *TOX*, *FAM110B*, and *SDCBP*. Similarly, Srikanth et al. (2020) reported that the most significant SNPs on chromosome 14 were located in *UBXN2B*, *CYP7A1*, *SDCBP*, and *TOX*, which have been regarded as positional candidate genes for carcass weight in Hanwoo cattle. It was also reported that *CYP7A1* and *SDCBP* are positional candidate genes for carcass weight and eye muscle area in Hanwoo cattle (Bhuiyan et al., 2018; Srivastava et al., 2020), weaning weight in Brangus cattle (Weng et al., 2016), and feed efficiency traits in Nellore cattle (Brunes et al., 2021). The *TOX* gene located within this region is associated with reproductive traits in Nellore (de Camargo et al., 2015), residual feed intake and mid-test metabolic weight in SimAngus (Seabury et al., 2017), and development of puberty in Brahman cattle (Fortes et al., 2012). In addition, the *UBXN2B* gene was found to be associated with mid-test metabolic weight in SimAngus (Seabury et al., 2017) and carcass weight, carcass fat, and carcass conformation in Simmental cattle (Purfield et al., 2019), which is known as a protein-coding gene involved in endoplasmic reticulum biogenesis. *FAM110B* has been reported to be associated with fat thickness in composite beef cattle (Roberts, 2018). This gene functions in the cell cycle and cell growth and might play an important role in increasing carcass weight in beef cattle by increasing cell number and size. A pleiotropic QTL on chromosome 6 at 38–39 Mb was associated with tenderloin, striploin, brisket, top round, bottom round, and shank, which explained the largest (6.46%) and smallest (2.7%) proportion of genomic variance for bottom round and tenderloin, respectively. This region harbors relevant candidate genes, including *PKD2*, *SPP1*, *MEPE*, *IBSP*, *LAP3*, *NCAPG*, and *LCORL*. Most of the positional genes detected on chromosome 6 have previously been associated with many economically important traits in beef and dairy cattle. In a study on Brangus beef cattle, Weng et al. (2016) reported that most positional genes associated with direct birth weight, weaning weight, and yearling weight are located on chromosome 6. Similarly, Saatchi et al. (2014) identified a large-effect pleiotropic QTL located on chromosome 6 at 37–42 Mb was associated with direct birth weight, calving ease, carcass weight, rib eye muscle area, and weaning weight across several cattle breeds. In addition, it has previously been reported that a large number of significant SNPs associated with skeleton trait in Simmental cattle that were harbored by *LAP3*, *FAM184B*, *LCORL*, and *NCAPG* genes (Xia et al., 2017). A major QTL on chromosome 6, extending from 36 to 39 Mb related to carcass weight, was also identified in Japanese Black cattle (Nishimura et al., 2012). Interestingly, the *NCAPG* and *LCORL* genes, while being associated with the skeletal type traits (Hoshiba et al., 2013; Doyle et al., 2020) have also been regarded as positional candidate genes for direct calving ease, feed intake, gain, meat, and carcass traits (Lindholm-Perry et al., 2011; Bongiorni et al., 2012; Purfield et al., 2019), as well as growth and lipid deposition (Snelling et al., 2010; Weikard et al., 2010) across several breeds. Moreover, Liu et al. (2015) identified *LCORL* as a positional gene related to weight and carcass composition traits in chickens based on GWAS and differentially expressed gene studies. Other notable positional candidate genes in this region including *PKD2*, *LAP3*, *SPP1*, *MEPE*, *IBSP* and *MED28,* have been associated with carcass weight and growth traits (Lindholm-Perry et al., 2011; Weng et al., 2016; Roberts, 2018; Naserkheil et al., 2020), milk production (Olsen et al., 2005), and reproductive traits and puberty (Daetwyler et al., 2008; Cánovas et al., 2014) in beef and dairy cattle.

The other large-effect pleiotropic QTL identified in multiple traits (tenderloin, sirloin, brisket, top round, bottom round, shank, and flank) was located on chromosome 19 at position 26–27 Mb. This region explained between 1 and 2.07% of the genomic variance across the traits of interest, and harbors candidate genes, including *PITPNM3*, *WSCD1*, *MIS12*, and *RABEP1*. A recent study on linear type traits conducted by Doyle et al. (2020b) identified *PITPNM3* as a potential gene for chest depth in Angus cattle. The *WSCD1* gene encodes a protein with sulfotransferase activity involved in glucose metabolism, and is associated with udder depth in dairy cattle (Li et al., 2021), feed efficiency and feeding behaviors in pigs (Guo et al., 2015), and body size in beef cattle (An et al., 2020). The *MIS12* and *RABEP1* genes have also been recently linked to milk production traits in dairy cattle (Cai et al., 2020; Li et al., 2021). Two other pleiotropic genomic windows were located on chromosome 11 at 21–22 Mb, and on chromosome 14 at position 6–7 Mb, which the proportion of genomic variance explained by these windows ranged from 1.13 to 1.69 and from 0.87 to 1.9, respectively. The region on chromosome 14 that was found to be associated with sirloin, brisket, bottom round, and shank had no positional candidate genes in cattle, whereas its orthologous region on human chromosome 8 containing *KHDRBS3* gene that may play a role as a negative regulator of cell growth and inhibition of cell proliferation (Ma et al., 2017). For QTL region on chromosome 11, a total of 15 genes annotated were found to be related to brisket, top round, bottom round, and shank. Overall, 11 chromosomal regions identified in this study were trait-specific QTLs for six traits. Three trait-specific QTLs were identified for tenderloin, which are distributed on chromosomes 4 at 77–78 Mb, 6 at 37–38 Mb, and 10 at 32–33 Mb. The trait-specific QTL on chromosome 6 was responsible for 2.74% of the genomic variance in tenderloin and harbors the positional candidate genes *PPM1K*, *ABCG2*, and *PIGY*. The *PPM1K* gene is involved in cellular survival, phosphorus metabolic process, amino acid dephosphorylation, and development by regulating mitochondrial permeability transition pore function, which have been shown to be associated with increased carcass weight, mid-point metabolic weight, reduction of residual feed intake, feed efficiency conversion ratio, and marbling score in crossbred beef cattle (McClure et al., 2010). The *ABCG2* gene is related to body weight (Weng et al., 2016) and milk yield and composition traits (Cohen-Zinder et al., 2005), which is involved in iron transport and metabolism. *PIGY* is a member of the PIG gene family, which encodes the glycosylphosphatidylinositol-N-acetylglucosaminyltransferase (GPI-GnT) complex and plays an important role in cell–cell interactions. A previous study reported that there were significant effects of copy number variation of the *PIGY* gene on growth traits in three Chinese sheep breeds (Feng et al., 2020). Brisket also had three trait-specific QTLs located on chromosomes 3 at 98–99, 10 at 49–50, and 25 at 32–33 Mb and explained 1.27, 0.92, and 0.92% of the genomic variance for this trait, respectively. The top round had two trait-specific QTLs. One was located on chromosome 4 at 6–7 Mb (GV% = 1.46), and the other on chromosome 25 at 4–5 Mb (GV% = 0.93). A QTL for the bottom round was identified on chromosome 16 at 25–26 Mb, which accounted for 1.13% of the genomic variance and harbors the positional candidate gene *DUSP10*. Previous studies have shown that the *DUSP10* gene is associated with growth traits (Ribeiro et al., 2021) and carcass weight (Chang et al., 2018) in beef cattle. The QTL located on chromosome 6 at position 39–40 Mb was associated with shank and had a greater proportion of genomic variance (4.89%) than other trait-specific QTLs. Only a QTL on chromosome 4 at 8–9 Mb, which was responsible for 3.23% of the genomic variance for the rib, was identified. The *CDK14* gene is located in this region and is associated with fatty acid profile (C18:1 *trans*-9) in the intramuscular fat of *Longissimus thoracis* muscle of Nellore cattle (Lemos et al., 2016). According to these results, most primal cut traits are probably controlled by several QTLs with large effects. Among these, genomic regions located on chromosomes 6 and 14 were considered as hot spots for several causal variants related to many economically important traits in beef cattle. A similar conclusion was given by Mehrban et al. (2021), who identified major QTLs on chromosomes 6 and 14 for EMA, yearling weight, and particularly for CW using the weighted single-step GWAS in Hanwoo cattle.

Gene ontology and pathway enrichment analyses were carried out to gain insight into the genes identified within QTL windows using g:Profiler and DAVID functional classification clustering tools (Table 3). Our analyses revealed the significant GO terms classified into the biological processes, cellular components, molecular functions, and seven KEGG pathways were enriched for the studied traits. It is interesting to note that the majority of the common genes identified for the primal cut traits are involved in growth-related processes: growth, positive regulation of growth, muscle structure development, regulation of cell development, muscle organ development, tissue development, skeletal system development, biomineral tissue development, and animal organ development. Among them, six genes, namely *HLX*, *SOS1*, *SDCBP*, *ANXA2*, *FZD1*, and *MEPE*, were highlighted as the main candidates for the traits under study in at least three biological pathways. The *HLX* gene, located on chromosome 16 at approximately 25–26 Mb, is a protein-coding gene that is involved in embryogenesis and hematopoiesis. It has also been shown to be associated with intramuscular fat in composite beef cattle (Roberts, 2018). The *SDCBP* gene is located on a conserved region on chromosome 14. It encodes a protein that binds to a variety of transmembrane proteins and plays a crucial role in carcass and meat quality traits. *ANXA2* gene is known to encode a member of a widely distributed, phospholipid-binding, calcium-regulated, peripheral membrane protein family known as annexins. This gene is involved in molecular functions related to calcium channel activity and lipid binding. It is not surprising then, that the role of calcium in meat tenderness and muscle contraction, and is a key regulator of muscle growth in beef cattle (Sadkowski et al., 2009). *ANXA2* knockout affects white adipose tissue hypotrophy due to reduced fatty acid uptake in mice (Salameh et al., 2016). Furthermore, this gene is associated with feed conversion efficiency in beef cattle (Al-Husseini et al., 2014). The GO terms related to the lipid metabolic process, cellular responses to the chemical stimulus, cell cycle, localization, and regulation of fat cell differentiation were also significantly represented in the pleiotropic QTL windows (Table 3). Pathway enrichment revealed that eight genes from six window regions (located on chromosomes 4, 6, 11, 14, and 25) were significantly associated with the metabolic pathway (bta01100). Among the genes harbored in this pathway, *CYP7A1* is involved in the transport, synthesis, and secretion of cholesterol, steroids, and other lipids (Zhao et al., 2013), which play a crucial role in digestion and absorption of dietary fat and contribute in maintaining the balance of cholesterol and lipid metabolism within the body (Monte et al., 2009). Interestingly, the extracellular matrix (ECM)-receptor interaction (bta04512) and focal adhesion (bta04510) pathways were enriched in the genes *SPP1* and *IBSP* from a pleiotropic QTL located on chromosome 6 at 38–39 Mb, which functions as a positive regulator in skeletal muscle cells and is involved in bone mineralization processes. The ECM-receptor interaction pathway plays a key role in tissue, organ morphogenesis, cell maintenance, and tissue structure and function (Mariman and Wang, 2010). It was previously reported that the ECM-receptor is upregulated in subcutaneous fat and intramuscular fat and appears to be involved in adipogenesis and meat tenderness (Taye et al., 2018). Focal adhesion also participates in important biological processes and serves as a mechanical link to ECM receptors and other molecules. These results will improve our understanding of enriched molecular processes, pathways, and genes associated with the primal cut traits and shed some light on how different pathways control these traits.

## Conclusion

In the current study, 16 genomic regions (SNP windows) were found to be associated with 10 primal cut traits in Hanwoo cattle using a single-step Bayesian regression GWAS. Within these windows, five QTLs had pleiotropic effects, with the most significant region located on chromosome 14 at position 26–27 Mb. Several candidate genes with potential functions in tissue development, regulation of growth, and lipid metabolism for the related traits were highlighted, among which *SPP1*, *IBSP*, *PKD2*, *SDCBP*, *PIGY*, *CYP7A1*, and *MEPE* were well-known. Moreover, our findings can contribute to a better understanding of the genetic basis and biological processes underlying the traits of interest; consequently, information on QTL regions can be used to search for causal mutations and marker-assisted or genomic selection in Hanwoo breeding schemes.

## Data Availability

The datasets presented in this study can be found in online repositories. The names of the repository/repositories and accession number(s) can be found below: [(https://www.hknu.ac.kr/dhlee/2797/subview), (https://www.aiak.or.kr) and (https://www.mtrace.go.kr)].

## Supplementary Material

Supplementary Figure S1 Location of 10 carcass primal cut yields in Hanwoo cattle.

Supplementary Figure S2 Manhattan plots of the percentage of additive genetic variance explained by 1-Mb windows for primal cut traits in Hanwoo cattle.

## Abbreviations

HIC, hanwoo improvement center; GWAS, genome-wide association study; ssGBLUP, single-step genomic best linear unbiased prediction; ssBR, single-step Bayesian regression; QTL, quantitative trait loci; SNP, single nucleotide polymorphism; BFT, backfat thickness; CW, carcass weight; EMA, eye muscle area; MS, marbling score; GO, gene ontology; KEGG, kyoto encyclopedia of genes and genomes; MCMC, markov chain monte carlo; GV, genomic variance; AGV, additive genetic variance; WPPA, window posterior probability of association.
