# Supplementary material for: Genome-wide Association Study for Carcass Primal Cut Yields Using Single-step Bayesian Approach in Hanwoo Cattle
Source: Front Genet. 2021 Nov 26;12:752424. doi: 10.3389/fgene.2021.752424 (PMC8662546; doi:10.3389/fgene.2021.752424)
Supplement: Supplementary file 1 [file Table1.docx]

**TABLE S1.** Estimates of additive genetic variance (σ^2^_u_), genomic variance (σ^2^_m_), residual variance (σ^2^_e_), and phenotypic variance (σ^2^_p_) for primal cut traits in Hanwoo cattle.

| Trait | σ^2^_u_ | σ^2^_m_ | σ^2^_e_ | σ^2^_p_ | σ^2^_m_/( σ^2^_a_+ σ^2^_m_) |
| --- | --- | --- | --- | --- | --- |
| Tenderloin | 0.05 (0.01) | 0.15 (0.01) | 0.23 (0.01) | 0.42 (0.01) | 0.75 |
| Sirloin | 2.05 (0.54) | 3.97 (0.31) | 6.39 (0.5) | 12.42 (0.33) | 0.66 |
| Striploin | 0.12 (0.03) | 0.26 (0.02) | 0.46 (0.03) | 0.85 (0.02) | 0.68 |
| Chuck | 0.80 (0.24) | 2.02 (0.17) | 6.05 (0.28) | 8.86 (0.23) | 0.72 |
| Brisket | 1.23 (0.29) | 2.65 (0.17) | 2.66 (0.26) | 6.54 (0.18) | 0.68 |
| Top round | 0.82 (0.18) | 1.78 (0.12) | 1.91 (0.16) | 4.51 (0.13) | 0.68 |
| Bottom round | 1.67 (0.40) | 4.89 (0.29) | 4.84 (0.38) | 11.40 (0.31) | 0.75 |
| Shank | 0.45 (0.10) | 0.97 (0.06) | 0.90 (0.09) | 2.32 (0.06) | 0.68 |
| Flank | 1.63 (0.48) | 4.25 (0.35) | 10.69 (0.55) | 16.58 (0.43) | 0.72 |
| Rib | 4.50 (1.38) | 10.62 (0.84) | 22.63 (1.4) | 37.76 (1.00) | 0.70 |

*The standard deviation (SD) of the parameters is indicated in the parentheses.
* Heritability (h^2^) is sum of σ^2^_u_ and σ^2^_m_ divided by σ^2^_p_ as mentioned in Table 1 in the revised manuscript.
