# Supplementary figures and images for: Genome-wide Association Study for Carcass Primal Cut Yields Using Single-step Bayesian Approach in Hanwoo Cattle
Source: Front Genet. 2021 Nov 26;12:752424. doi: 10.3389/fgene.2021.752424 (PMC8662546; doi:10.3389/fgene.2021.752424)

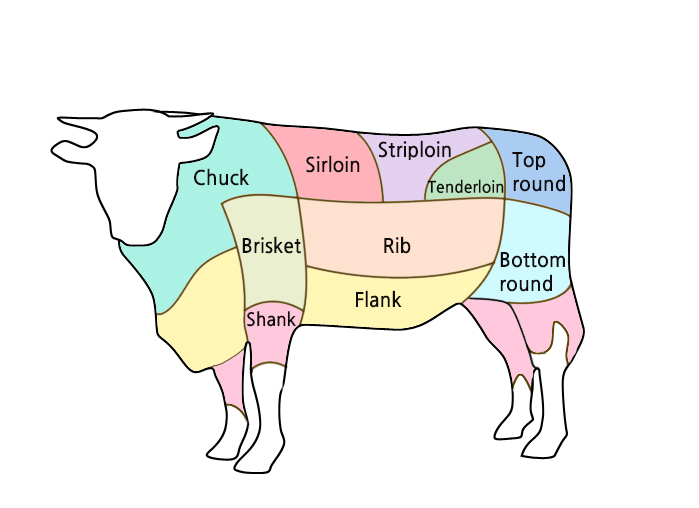

Supplement: Supplementary file 2 [file Image1.JPEG]

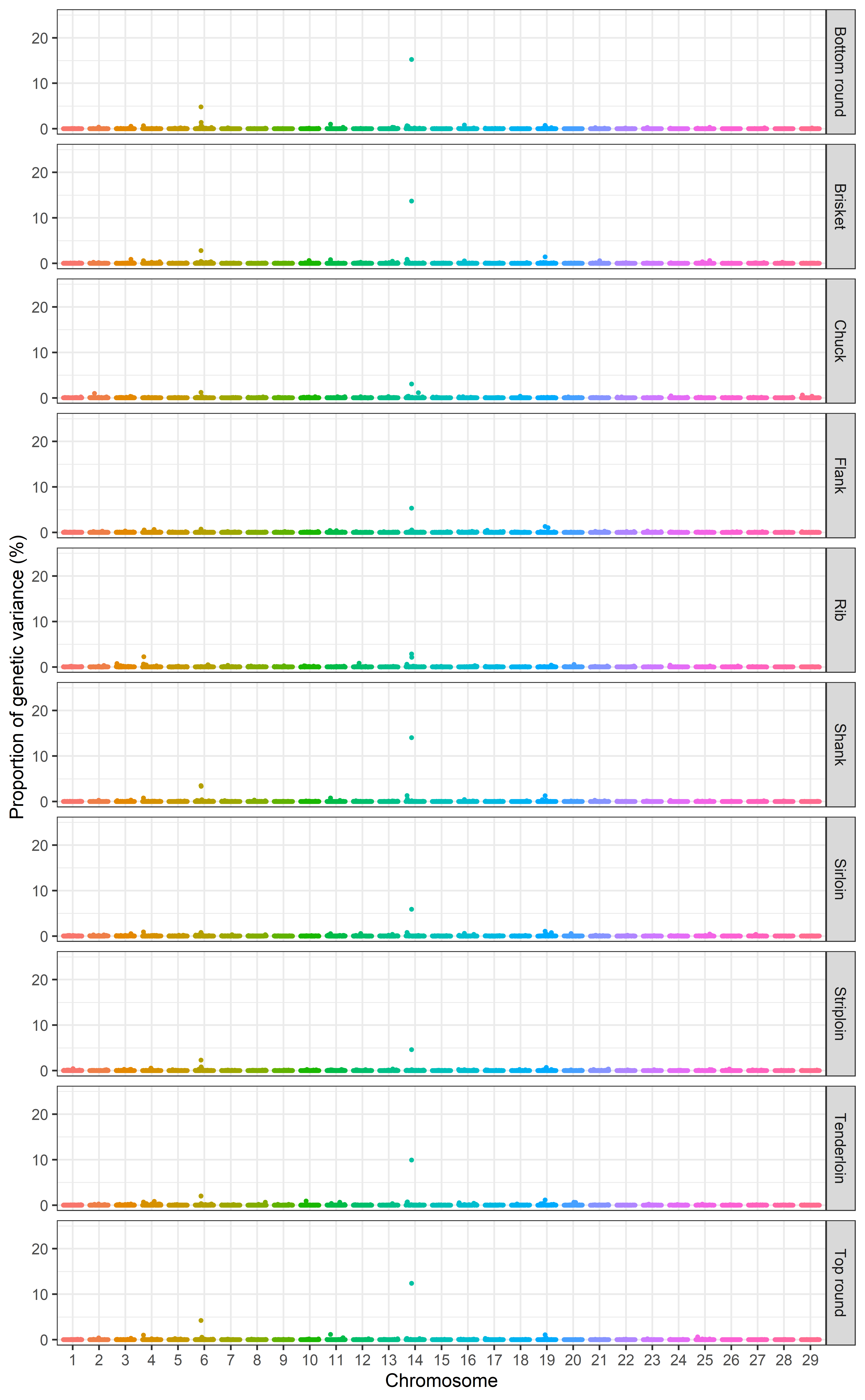

Supplement: Supplementary file 3 [file Image2.TIFF]
